# Continuing Education for Gerontological Social Work: Findings From Post-Graduate Advanced Practice Training

**DOI:** 10.1093/geroni/igaa057.038

**Published:** 2020-12-16

**Authors:** Raza Mirza, Alison Kilbourn, Samir Sinha, Jessica Hsieh

**Affiliations:** 1 National Initiative for the Care of the Elderly, Toronto, Ontario, Canada; 2 Sinai Health, Toronto, Canada; 3 University of Toronto, Toronto, Ontario, Canada

## Abstract

The social work profession aims to help all individuals, families, and communities enhance their overall well-being. While gerontological social workers primarily work with seniors, they are often tasked with addressing the needs of not only their senior client, but the client’s network as well. Social workers are also asked to stay current with respect to new legislation, policy, and systemic changes to help their clients. Thus, gerontological social workers often need to obtain advanced practice education in a number of areas, as related to gerontology. Responding to this gap, the National Initiative for the Care of the Elderly (NICE) and Sinai Health offered a series of innovative advanced practice gerontological social work courses - hybrid online and in-class - to those looking to improve their knowledge and competencies in the following five identified 'high impact' areas: (1) Medical Assistance in Dying (MAiD); (2) cultural competence and LGBTQI2S; (3) mental health; (4) legal issues and aging; (5) dementia. Participants in the courses completed pre- and post-surveys assessing knowledge, attitudes and competencies with the subject matter, with responses helping to improve understanding of how to provide the most appropriate resources for those who care for older adults and how to better shape future education/training programs. Findings suggest that gerontological social workers may benefit from 'tailored refresher courses' that bridge knowledge and practice gaps, that the optimal update time may be every three years, and that clinicians benefit from being trained by interdisciplinary teams, rather than by someone of the same profession.

